# Analysis of chicken anemia virus genome: evidence of intersubtype recombination

**DOI:** 10.1186/1743-422X-8-512

**Published:** 2011-11-10

**Authors:** Yassir M Eltahir, Kun Qian, Wenjie Jin, Aijian Qin

**Affiliations:** 1 Ministry of Education Key Lab for Avian Preventive Medicine, Yangzhou University, Yangzhou, 225009, PR China; 2 Key Laboratory of Jiangsu Preventive Veterinary Medicine, Yangzhou University, 225009, PR China; 3 Department of Preventive Medicine and Veterinary Public Health, Faculty of Veterinary Science, University of Nyala, Nyala, Sudan

## Abstract

**Background:**

Chicken anemia virus (CAV) is the causative agent of chicken infectious anemia. CAV putative intergenotypic recombinants have been reported previously. This fact is based on the previous classification of CAV sequences into three genotypes. However, it is unknown whether intersubtype recombination occurs between the recently reported four CAV genotypes and five subtypes of genome sequences.

**Results:**

Phylogenetic analysis, together with a variety of computational recombination detection algorithms, was used to investigate CAV approximately full genomes. Statistically significant evidence of intersubtype recombination was detected in the parent-like and two putative CAV recombinant sequences. This event was shown to occur between CAV subgroup A1 and A2 sequences in the phylogenetic trees.

**Conclusions:**

We revealed that intersubtype recombination in CAV genome sequences played a role in generating genetic diversity within the natural population of CAV.

## Background

Chicken anemia virus (CAV) was first reported in 1979 in specific-pathogen-free (SPF) chickens [1]. CAV belongs to the Circoviridae and is a non-enveloped, icosahedral virus with a negative-sense, single-stranded circular DNA. The viral genome consists of 2.3 kb, with three partially overlapping open reading frames. CAV infection is an economically important clinical and subclinical disease in broiler chickens, with a worldwide distribution [2].

CAV isolates show extremely limited genetic variability worldwide [3]. All isolates of CAV are suspected to belong to a single serotype [4]. Little is known about CAV genome recombination analysis. CAV putative intergenotype recombinants have been reported to occurs in the virus gene VP1 and results in a new virus genotype [5].

Recently, after adding more CAV approximately full genome sequences to GenBank, CAV sequences arising from different parts of world have been categorized into four genotype groups (A-D) and five subtypes (A1, A2, A3, D1 and D2) [6]. Therefore, the necessity of exploring these CAV genotypes for evidence of recombination as an important tool for genetic variability has been raised, to establish if any would help in understanding the evolutionary process in the CAV genome.

Here, we report evidence of intersubtype recombination based on sequence analysis of the entire coding regions (VP1, VP2 and VP3) of CAV genomes.

## Methods

### Samples

DNA extraction, PCR screening, amplification of CAV genome, cloning and sequencing were carried out as previously described, briefly, primers VP1F: 5'AGCCGACCCCGAACCGCAAGAA'3 and VP1R: 5' TCA GGG CTG CGT CCC CCA GTA CA'3 were used to amplify the VP1 region and primers VP2F: 5' GCG CAC ATA CCG GTC GGC AGT'3 and VP2R: 5' GGG GTT CGG CAG CCT CAC ACT AT'3 were used to amplify the VP2 region [6] for 10 spleen samples collected between April 2010 and December 2010 at Yangzhou University Veterinary Hospital during necropsy. Samples originated from different commercial chicken farms in Anhui (n = 3) and Jiangsu (n = 7) provinces. For each samples, DNA extraction and PCR was run at least twice. Animal experiments were conducted in accordance with the guidelines provided by the Chinese Council on Animal Care. All experiments complied with institutional animal care guidelines and were approved by University of Yangzhou Animal Care Committee (protocol number 06R015).

### Sequence analysis

To look for recombination in CAV sequences, we used 55 published full genomes sequences that are currently available in GenBank, together with 10 sequences characterized in the present study. Multiple alignments of either CAV full or subgenomic regions were performed and analyzed using ClustalW [7]. Unrooted phylogenetic trees were constructed using the neighbor-joining (NJ) method, and visualized and edited using MEGA 3.1 software [8]. The evolutionary distances were estimated using the Kimura two-parameter method. Bootstrap analyses were performed with 1,000 repeat samples of the data sets. To reduce redundancy, isolates with previous reports of intergenotypic recombination [5] were excluded from analysis.

### Identification of recombination

Recombination breakpoint events in the multiple alignments were detected with the Recombination Detection Program 3 (RDP3), using the automated suite of algorithms implemented in the RDP3 with default settings. These included RDP, GENECONV, BootScan, MaxChi, Chimaera, SiScan, Phyl- Pro, LARD, and 3Seq [9,10]. For the RDP algorithm, the reference sequence parameter (internal and external reference) was used as recommended by the manual. The multiple-aligned sequences were cut into sections at the positions where the putative recombination breakpoints were identified, and phylogenetic analyses were carried out by MEGA 3.1 software [8]. Confirmation of recombination break points was further carried out by bootscanning using SimPlot [11].

## Results

### Sequences accession numbers

CAV genome sequences characterized in this study were submitted to GenBank with accession numbers [GenBank: FR 850021, GenBank: FR 850022, GenBank: FR 850023, GenBank: FR 850024, GenBank: FR 850025, GenBank: FR 850026, GenBank: FR 850027, GenBank: FR 850028, GenBank: FR 850029, GenBank: FR 850030].

### Phylogenetic tree reconstructions

**Figure 1:**
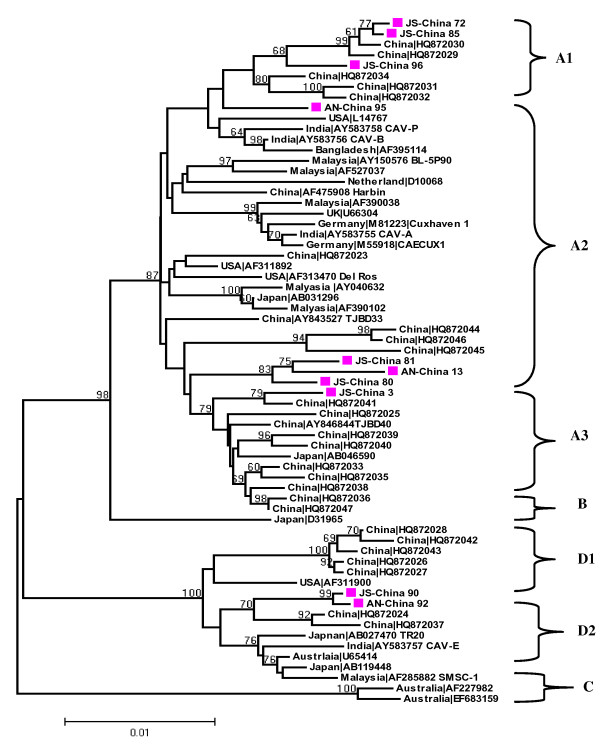
**Phylogenetic analysis of the nucleic acid sequence of the 10 new complete VP1, VP2 and VP3 sequences from Anhui (AN) and Jiangsu (JS) provinces, China, and the 55 relevant VP1, VP2 and VP3 sequences currently available in GenBank**. Values ≥ 70 are indicated on the branches (as percentages). Sequences from the present study (colored closed symbols) are named as PP-China, where PP is the area of origin. Sequences from GenBank were given the country name followed by accession number. The four major groups were identified as A, B, C and D.

When an NJ phylogenetic tree was reconstructed, we found that the high number of sequences included maintained the four genotype groups (A-D) and five subgroups (A1, A2, A3, D1 and D2) topology that was present in previously published trees (Figure 1)[6]. When the RDP, GENECONV, Boot Scan, MaxChi, Chimaera, SiScan, Phyl-Pro, LARD, and 3Seq algorithms were used to detect recombination in CAV whole genome sequences, only one significant recombination event was detected. For this event, seven out of nine algorithms detected significant recombination at the same location in the CAV genome with p values ranging from[6.215 × 10^-07 ^to 4.544 × 10^-2 ^] (Table 1). The location of two significant break points was in the VP1 coding region (*nt *positions 768 and 1286) of AN-China 13, which was considered as the daughter or recombinant, with the major parent being the HQ872045 and the minor parent being JS-China 72.

**Table 1:** Recombination Statistics

Algorithm	Recombination P-Value	nt Position
RDP	4.544 × 10^-02^	768-1286
GENECONV	n/a	n/a
BootScan	1.179 × 10-^02^	768-1286
MAXcHI	4.692 × 10^-03^	768-1286
Chimaera	1.768 × 10^-04^	768-1286
SiScan	6.215 × 10^-07^	768-1286
PhylPro	n/a	n/a
LARD	1.499 × 10^-05^	768-1286
3Seq	1.612 × 10^-05^	768-1286

### Confirmation of recombination event

**Figure 2:**
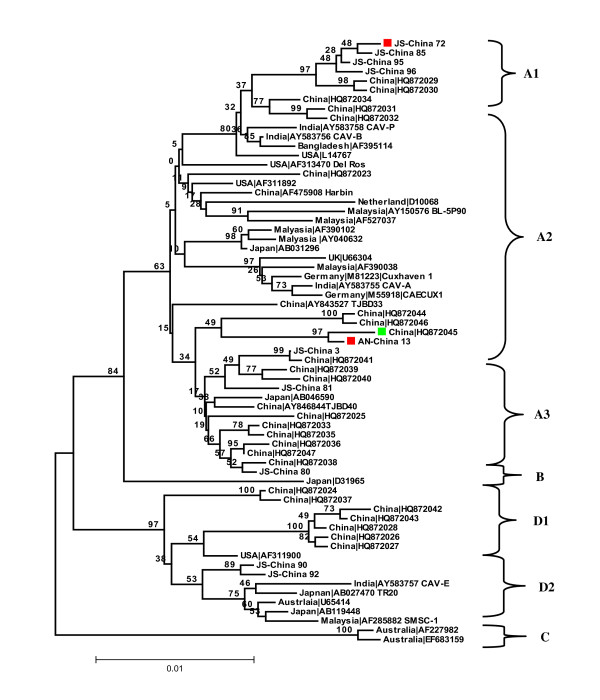
**Phylogenetic analysis of the non-recombinant region of the 10 new CAV sequences from Anhui (AN) and Jiangsu (JS) provinces, China, and the 55 relevant CAV sequences currently available in GenBank**. Sequences from the present study are named as PP-China, where PP is the area of origin. Red and green colored closed symbols represent recombinant, minor parent and major parent sequences respectively. GenBank sequences were given the country name followed by accession number. The four major groups were identified as A, B, C and D.

**Figure 3:**
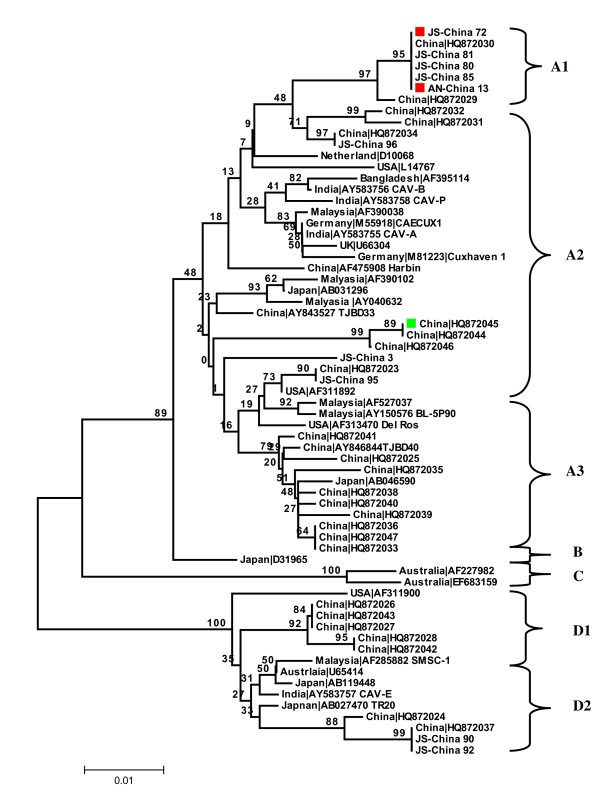
**Phylogenetic analysis of the recombinant region of the 10 new CAV sequences from Anhui (AN) and Jiangsu (JS) provinces, China, and the 55 relevant CAV sequences currently available in GenBank**. Sequences from the present study are named as PP-China, where PP is the area of origin. Red and green colored closed symbols represent recombinant, minor parent and major parent sequences respectively. GenBank sequences were given the country name followed by accession number. The four major groups were identified as A, B, C and D.

**Figure 4:**
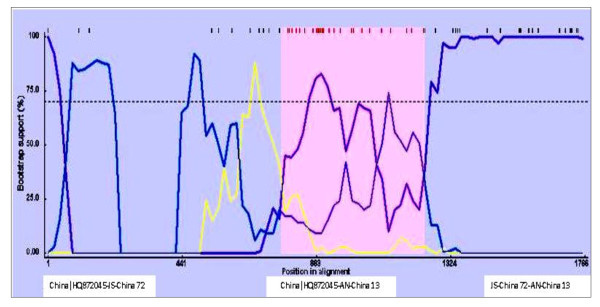
**Bootscan analysis of the recombinant, major parent and the minor parent sequences**. Bootscan was based on pairwise distance model with the window size 200, step size 50, and 1000 bootstrap replicates generated by the RDP3 program.

**Figure 5:**
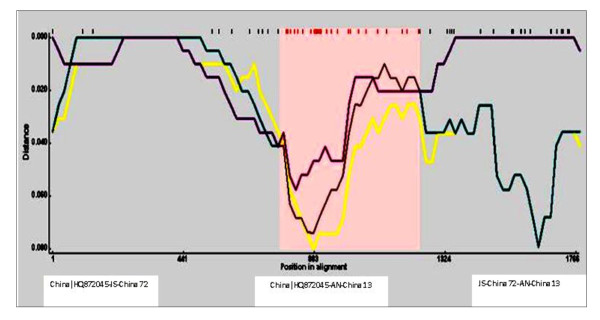
**Distance plot analysis of the recombinant, major parent and the minor parent sequences**. Pair-wise distances for distance plot were calculated for each window using DNADIST.

The region identified as containing the recombination breakpoint was confirmed by comparing the phylogenetic tree topologies of the entire CAV coding region without the recombinant region. The tree topology was essentially the same as for the whole genome. For the recombinant region, changes in tree topology and decreases in distance that separates the daughter (AN-China 13) and minor parent (JS-China 72) sequences were observed. AN-China 13 was located in subtype A2 in the tree constructed with the non-recombinant region (Figure 2), and was found within subtype A1 when the tree was developed with the recombinant region (Figure 3). Furthermore, when the recombinant minor parent and major parent sequences were further analyzed, recombination was supplemented and supported by identifying the two points of crossover by Boot Scan and Distance plot (Figure 4, 5). In addition, high similarity (99.3%) between AN-China 13 and JS-China 72 was observed.

## Discussion

Recombination is one of the evolutionary processes that shape the architecture of viral genomes. Ignoring the occurrence of recombination may influence the analysis of genetic data and the conclusions derived from it. Recombination analysis of CAV genomes is very limited. CAV intergenotype recombination break points in the VP1 region have been reported in only a single study using 35 CAV full genomes sequences [5]. However, to date, there is no evidence of inter- or intrasubtype recombination between CAV genomes, to the best of our knowledge. Therefore, the present study investigated CAV whole genome sequences to explore the effect of recombination in the genetic variation of CAV sequences.

Here, we report the first evidence of intersubtype recombination events in CAV genome sequences amplified from two different areas (Anhui and Jiangsu provinces) in China. Intersubtype mosaic viruses are the results of co-infection events in geographic regions where multiple subtypes circulate [12,13]. Similarly, co-infection with CAV that has been documented by sequencing electropherogram double peaks and cloning has also been reported [14]. Moreover, intersubtype recombination in other virus subtypes has also been reported [15,16].

Natural recombination in the VP1 of the CAV genome, with a resultant new genotype, has suggested speeding up of CAV evolution [5]. However, it should be noted that these findings were based on a limited number of CAV sequences and the previous categorization of CAV sequences into three genotypes. In contrast, despite adding more sequences here, we confirmed that CAV genomes can be categorized into their recent four genotype groups (A-D) and five subtypes (A1, A2, A3, D1 and D2) [6].

The recombination breakpoints detected in CAV subgroup A1 and A2 sequences in the present study were also found in the VP1 protein. This resulted in the sequence from subgroup A2 being located within subgroup A1. It is known that most nucleotide variation in the CAV genome is observed in VP1, in addition to the fact that virulence of CAV is also determined by VP1 [17,18]. The cumulative evidence of recombination breakpoints in the VP1 of the CAV genome may be indicative of favorable selection for recombination in this variable region. Sequence variations at specific sites between HIV-1 isolates have been reported to introduce unique recombination hotspots and increase recombination frequencies in the C3/V4 env region [19]. However, whether there is correlation between the highest genetic variability in CAV genomes observed in the VP1 and recombination still needs to be investigated.

## Conclusions

Using bioinformatics analysis, we were able to detect only a single incidence of CAV intersubtype recombination that contributed to CAV genetic variation. More detailed analysis for further CAV intragenotypic recombination is still required.

## Competing interests

The authors declare that they have no competing interests.

## Authors' contributions

YME designed the study, carried out the experiments, analyzed the data, and drafted the manuscript. QA supervised all the experiments and participated in the data analysis. JW and QK discussed and prepared the final report. All authors have read and approved the final manuscript.
